# The association between delusional-like experiences, and tobacco, alcohol or cannabis use: a nationwide population-based survey

**DOI:** 10.1186/1471-244X-11-202

**Published:** 2011-12-28

**Authors:** Sukanta Saha, James G Scott, Daniel Varghese, Louisa Degenhardt, Tim Slade, John J McGrath

**Affiliations:** 1 Queensland Centre for Mental Health Research, The Park Centre for Mental Health, Wacol, QLD 4076, Australia; 2 Metro North Mental Health, Royal Brisbane and Women's Hospital, Brisbane, QLD, Australia; 3 The University of Queensland Centre for Clinical Research, Brisbane, QLD, Australia; 4 Department of Psychiatry, University of Queensland, St Lucia QLD, Australia; 5 Princess Alexandra Hospital, Woolloongabba, QLD 4102 Australia; 6 National Drug & Alcohol Research Centre, University of New South Wales, NSW, Australia; 7 Queensland Brain Institute, University of Queensland, St Lucia, QLD, Australia

**Keywords:** Delusional-like experiences, smoking, cannabis, alcohol use or dependence

## Abstract

**Background:**

Previous population-based studies have found that delusional-like experiences (DLE) are prevalent in the community, and are associated with a wide range of mental health disorders including substance use. The aim of the study was to explore the association between DLE and three commonly used substances - tobacco, alcohol and cannabis.

**Methods:**

Subjects were drawn from the Australian National Survey of Mental Health and Wellbeing 2007. The Composite International Diagnostic Interview was used to identify DLE, common psychiatric disorders, and substance use. We examined the relationship between the variables of interest using logistic regression, adjusting for potential confounding factors.

**Results:**

Of 8 773 participants, 8.4% (n = 776) subjects endorsed one or more DLE. With respect to tobacco use, compared to nonusers, DLE were more common in those who (a) had daily use, (b) commenced usage aged 15 years or less, and (c) those who smoked heavily (23 or more cigarettes per day). Participants with cannabis use disorders were more likely to endorse DLE; this association was most prominent in those with an onset of 16 years or younger. In contrast, the pattern of association between DLE versus alcohol use or dependence was less consistent, however those with early onset alcohol use disorders were more likely to endorse DLE probe items.

**Conclusions:**

While cannabis use disorders have been previously linked with DLE, our findings linking alcohol and tobacco use and DLE suggest that the influence of these substances on psychosis-related outcomes warrants closer scrutiny in longitudinal prospective studies.

## Background

In recent decades, there has been intense interest in the links between cannabis use and psychosis [1,2]. Several prospective population-based studies have found that cannabis use is associated with an increased risk of developing psychotic disorders later in life [3-8]. Among those who have developed psychotic disorders, cannabis use is associated with an earlier age of onset of such disorders [9]. A growing body of evidence also shows that cannabis use is associated with attenuated features of psychoses, known as psychotic-like experiences (PLE) or delusional-like experiences (DLE) [10-15]. In particular, population-based studies in adolescents have reported that early onset cannabis use is associated with a greater likelihood of DLE endorsement [11,16,17].

Apart from cannabis, there has been less study of the links between other substances and risk of psychosis or DLE. Several prospective studies have reported that tobacco smoking was associated with an increased risk of later psychosis, and there was a dose-response relationship between the variables of interest [18-20]. With respect to alcohol, one study found that younger age at onset of alcohol dependence was associated with increased risk of psychotic symptoms [21]. An Australian population-based study examined past-year associations between DLE, and the use of tobacco, cannabis and alcohol [15]. This study found that regular tobacco, alcohol and cannabis use disorders were much more common in those who screened positive for psychotic disorders (i.e. those who endorsed at least several CIDI items related to psychotic symptoms).

The previous Australian study [15] was unable to explore potential associations with age of onset of drug use and DLE. We had the opportunity to address this issue and explore the associations between DLE and commonly used substances such as tobacco, alcohol and cannabis. We were interested in exploring the association between the variables of interest when adjusted for a range of potential confounds. Based on the existing literature, we predicted that tobacco use, cannabis and alcohol use disorders would be associated with endorsement of DLE. We also predicted that those who reported an earlier age of first use for these substances, or those who had greater intake, would be more like to endorse DLE compared to non-users.

## Methods

### Participants

Subjects were drawn from the Australian National Survey of Mental Health and Wellbeing 2007 (NSMHWB). Details of the methodology have been published elsewhere [22]. In brief, the NSMHWB was a national face-to-face household survey of community residents aged between 16 and 85 years. Sampling was based on random selection from a stratified, multistage area probability sample of private dwellings. Interviews were carried out by trained interviewers from the Australian Bureau of Statistics, a statutory body responsible for conducting such surveys using ethical protocols that include written informed consent. In total, 8,841 individuals participated in the survey that represents an estimated population of 16 015 000 adults. As the response rate for this survey was lower than expected (60%), extensive non-response analyses were undertaken by the Australian Bureau of Statistics in order to assess the reliability of the survey estimates. As a result, adjustments were made to the weighting strategy (full details available elsewhere http://www.abs.gov.au[23]).

### Assessment of DSM-IV diagnoses, delusional-like experiences and physical disorders

A modified version of the World Mental Health Survey Initiative of the Composite International Diagnostic Interview (WMH-CIDI 3.0) was used to generate lifetime presence of DLEs, and DSM IV based diagnoses of a wide range of common mental health disorders including anxiety disorders, depressive disorders, and alcohol or drug abuse or dependence.

For the assessment of DLEs, we used the items in Section G designed to screen for possible psychosis. Details of the DLEs are given in Table 1. Briefly, the DLE component was composed of three 'screen' items followed by three 'probe' items. Subjects who responded positively to any of the screen items, were administered the 'probe' items. The items covered the following features of psychotic disorders: delusions of control, thought interference and passivity (Question 1 and 1a); delusions of reference and persecution (Question 2 and 2a); and grandiose delusions (Question 3 and 3a). While endorsement of screen items are more common, subsequent endorsement of probe items is more likely to be predictive of true psychotic features [24].

**Table 1:** CIDI Screen and Probes items for Psychosis (Delusional-like experiences, DLE)

Item G1 (PS1*):
Have you ever felt that your thoughts were being directly interfered with or controlled by another person?
***If yes, PS1A^#^:***
Did it come about in a way that many people would find hard to believe, for instance, through telepathy?
Item G2 (PS2*):
Have you ever had a feeling that people were too interested in you?
***If yes, PS2A^#^: ***
Have you had a feeling that things were arranged so as to have a special meaning for you, or even that harm might come to you?
Item G1 (PS3*):
Do you ever have any special powers that most people lack?
***If yes, PS3A^#^: ***
Do you belong to a group of people who also have these powers?
Item PS4^@^: Has a doctor ever told you that you may have schizophrenia?

*Screen items (lifetime) with answer (Yes/No): 'Any screen' items required 'Yes' answers to all three questions.

#Probe items (lifetime) with answer (Yes/No): 'Any probe' items required 'Yes' answers to PS1A and PS2A, and 'No' answer to PS3A.

^@^Sample excluded from the analyses (n = 68)

In order to keep a focus on isolated (subclinical) DLE, and in order to allow comparisons with our previous analyses based on this survey [25-28], individuals who screened positively for schizophrenia (i.e. respondents who reported 'Yes' to the item "*Had been told at any time by a psychiatrist that they had schizophrenia*") were excluded from the analyses (n = 68), leaving a total of 8 773 subjects for this study.

The WMH-CIDI instrument also includes checklists related to the presence of physical disorders. Details of the methodology have been published elsewhere [26]. Respondents were asked if they ever had any of six physical illnesses: (a) asthma, (b) gout, rheumatism or arthritis, (c) cancers, (d) diabetes or high blood sugar levels, (e) any heart attack, angina or high blood pressure, and (f) stroke or effects of stroke.

### Assessment of tobacco, cannabis and alcohol exposure

Respondents were asked whether they used tobacco regularly ("current user"), and their age of starting use, and current number of cigarettes or other tobacco products used per day. All persons were assessed for alcohol, and cannabis use disorders, based on DSM-IV diagnoses. Details of DSM-IV diagnoses for alcohol use disorders have been published elsewhere [29]. Briefly, in assessing the presence of DSM-IV alcohol use disorders, participants were initially asked whether they had consumed at least 12 alcohol drinks (containing 10 g of ethanol) in any one year in their life-time. Respondents who responded 'yes' were also asked whether they had ever in their lifetime drank on > = 3 days a week and/or have usually consumed > = 3 drinks on the days they were drinking. Only those who answered this question positively were administered the symptom questions that operationalise the DSM-IV alcohol use/dependence disorder diagnostic criteria. A series of 18 questions operationalised the four alcohol use (major role, legal, hazardous use, and social), and seven alcohol dependence (tolerance, withdrawal, cutdown, larger, time spent, give up and continued use) criteria.

Similar to the assessment of alcohol use disorder, the 2007 NSMHWB collected data on cannabis use disorders. In assessing for a cannabis use disorder, subjects were screened for any lifetime use - those who reported lifetime use were then asked a series of five questions for the diagnosis of use disorders, and 10 questions to establish lifetime diagnosis of dependence.

### Data analysis

For the main analyses, we examined the association between reporting any DLE (i.e. at least one of the G screen or probe items endorsed), and the tobacco, cannabis and alcohol related variables. For tobacco users, respondents were asked about (a) age at first use, and (b) current number of smokes per day. We divided responses into quartiles (lowest quartile indicative of earlier age of onset, or highest number of cigarettes use respectively). 'No smoking' was used as the *reference *group for both variables. For cannabis and alcohol, 'age of onset of use disorder' variables were also divided into quartiles where 'no onset' was used as the *reference *group.

Based on previous research of factors associated with DLE, in Model 1, we adjusted for sex and age of assessment [30,31]. As previous studies have reported DLEs to be associated with anxiety and depressive disorders [28], and physical disorders [25] we examined a second model adjusting for these potential confounding factors together with other demographic variables including marital status, migrant status, employment status and educational status. As many individuals use more than one substance [15], in Model 2 we also included adjustment for exposure to 'any other' drug or alcohol abuse/dependence (tobacco, alcohol, cannabis, opioid, stimulant and sedatives).

The sample was weighted to adjust for differential probabilities of selection within households, over-sampling of population subgroups and non-response to match census population distribution on a number of geographic and socio-demographic variables [22]. The initial weights were calibrated against known population estimates. Replicate weight variables were developed using the Jack-knife procedure of replication (i.e., the analysis was repeated after one subject was dropped and then the standard error was derived from the distribution of results from all ''minus one'' resamples) [32]. Analyses were performed using Proc *Surveylogistic *[33] which is designed to analyse complex survey sample using SAS (version 9.2;Cary, NC: SAS Institute). Chi-square tests for linear trend were used to assess dose-response relationships between the exposure variables and DLE.

## Results

Of the full sample, 49.6% were male, 57.0% were either married or in a de facto relationship, 32.2% had post-school qualification, 65.2% were employed, and 62.9% were born in Australia. Of the 8 773 subjects included in the study, 776 (8.4%) positively endorsed one or more DLE screen items (Table 2), and 295 (3.0%) endorsed one or more probe items.

**Table 2:** Descriptive statistics of delusional-like experiences (DLE), tobacco use, and cannabis and alcohol use disorders (n = 8,773*)

Exposure	Sample Number (%)	Delusional-like experiences endorsement	
		No (%)	Yes (%)^@^
Total sample	8773 (100.00)	7997(91.53)	776 (8.47)
			
Everyday tobacco use	1539 (17.97)	1325 (15.64)	214 (13.01)
Weekly tobacco use	302 (4.04)	266 (3.56)	36 (14.72)
No tobacco use	6932 (77.98)	6406 (72.33)	526 (7.24)
			
Cannabis use disorder, Lifetime	339 (4.22)	287 (3.63)	52 (13.98)
No Cannabis use disorder	8434 (95.78)	7710 (87.89)	724 (8.23)
			
Cannabis dependence disorder, Lifetime	130 (1.74)	99 (1.31)	31 (24.65)
No Cannabis dependence disorder	8643 (98.26)	7898 (90.21)	745 (8.19)
			
Alcohol use disorder, Lifetime	1490 (18.31)	1333 (16.60)	157 (9.35)
No alcohol use disorder	7283 (81.69)	6664 (74.93)	619 (8.28)
			
Alcohol dependence disorder, Lifetime	329 (3.71)	249 (2.84)	80 (23.42)
No alcohol dependence disorder	8444 (96.29)	7748 (88.69)	249 (7.89)

*The sample excludes the item related to past history of schizophrenia 'doctor ever told you that you have schizophrenia'; DLE (Screen items)

^@ ^Percentages denote risk set

### Association between tobacco use and DLE

About one-sixth (17.97%) of the population currently used tobacco every day (Table 3). Compared to those who did not use tobacco, individuals who smoked everyday were significantly more likely to endorse both DLE screen and probe items (Table 3). Individuals who first used tobacco at 15 years or younger were significantly more likely to endorse DLE. With respect to the number of cigarettes per day, individuals with heavy smoking (more than 22 cigarettes per day) were more likely to endorse DLE probe items. However, the pattern of findings in Model 2 did not suggest a clear dose-response relationship.

**Table 3:** Relationship between tobacco use, and delusional-like experiences (n = 8,773)

		Any Delusional-like experiences			
		Screen items		Probe items	
A. Tobacco use					
	Count (%)	Model 1^1^ OR^4 ^(95% CI^5^)	Model 2^2^ OR^4 ^(95% CI^5^)	Model 1^1^ OR^4 ^(95% CI^5^)	Model 2^2^ OR^4 ^(95% CI^5^)
**Smoking**					
Everyday	1539 (17.97)	1.86 (1.43, 2.43)*	1.39 (1.07, 1.81)*	2.37 (1.65, 3.39)*	1.66 (1.13, 2.44)*
Weekly	302 (4.05)	1.73 (0.99, 3.02)	1.28 (0.67, 2.45)	2.98 (1.09, 8.19)*	2.12 (0.65, 6.91)
Never	6932 (77.98)	***Reference***	***Reference***	***Reference***	***Reference***
**B. Age of start of smoking everyday**					
Very early start (Q1: 6-15 y)	517 (5.78)	**2.26 (1.63, 3.13)**	**1.61 (1.09, 2.36)**	**2.84 (1.82, 4.45)**	**1.82 (1.11, 3.00)**
Early start (Q2:16-17y)	352 (4.51)	**1.77 (1.18, 2.66)**	1.42 (0.92, 2.18)	**2.45 (1.31, 4.59)**	1.78 (0.89, 3.52)
Mid age start (Q3: 18-19y)	307 (3.56)	1.00 (0.59, 1.69)	0.79 (0.47, 1.36)	1.51 (0.69, 3.29)	1.19 (0.55, 2.61)
Late start (Q4: 20y+)	360 (4.10)	**1.93 (1.19, 3.12)**	1.56 (0.97, 2.51)	1.56 (0.83, 2.94)	1.13 (0.61, 2.12)
Not smoking at all	7234 (82.04)	***Reference***	***Reference***	***Reference***	***Reference***
**C. Number of cigarettes or tobacco products per day**					
Very heavy smoker (Q1: 23-75)	325 (3.97)	**2.19 (1.46, 3.28)**	**1.63 (1.06, 2.51)**	**2.35 (1.24, 4.44)**	1.54 (0.77, 3.09)
Heavy smoker (Q2:16-22)	313 (3.24)	**1.76 (1.07, 2.91)**	1.25 (0.76, 2.07)	**2.71 (1.60, 4.59)**	**1.77 (1.05, 2.99)**
Light smoker (Q3: 11-15)	314 (3.80)	**1.65 (1.03, 2.67)**	1.33 (0.82, 2.15)	1.72 (0.74, 4.01)	1.25 (0.52, 3.03)
Very light smoker (Q4: < 10)	584 (6.94)	**1.68 (1.13, 2.51)**	1.32 (0.85, 2.05)	**2.06 (1.24, 3.41)**	1.52 (0.89, 2.57)
Not smoking at all	7234 (82.04)	***Reference***	***Reference***	***Reference***	***Reference***

^1^Model 1 = Adjusted for age and sex; ^2^Model 2 = Adjusted for age, sex, marital status, migrant status, employment status, educational status, any anxiety disorders, any depressive disorders, physical disorders and 'other' harmful substance use/dependence ('other' harmful substance use/dependence denotes all other drug/alcohol use/dependence); ^4^OR = Odds Ratio, ^5^CI = 95% Confidence Interval; *significance: *p *< 0.01 (shown in bold)

### Association between cannabis use disorders and DLE

Table 4 shows the association between cannabis use disorders and DLE. Over 4% of the Australian adult population met criteria for lifetime cannabis use disorder. Those with cannabis dependence disorder were significantly more likely to endorse DLE. Individuals with (a) age of first use 16 years or younger were three times more likely to endorse DLE items. Compared to nonusers, users with the youngest age of onset (16 years or less) had about ten-fold increased odds of endorse DLE probe items.

**Table 4:** Relationship between lifetime cannabis use/dependence (meeting criteria for DSM-IV disorder) and delusional-like experiences (n = 8,773)

		Any Delusional-like experiences			
		Screen items		Probe items	
A. Lifetime cannabis use/dependence disorder					
	Count (%)	Model 1^1^ OR^4 ^(95% CI^5^)	Model 2^2^ OR^4 ^(95% CI^5^)	Model 1^1^ OR^4 ^(95% CI^5^)	Model 2^2^ OR^4 ^(95% CI^5^)
**Cannabis use disorder Cannabis dependence disorder**	339 (4.23) 130 (1.74)	1.73 (1.12, 2.66)* 3.53 (1.87, 6.66)*	1.13 (0.71, 1.82) 1.76 (1.00, 2.75)*	2.28 (1.27, 4.12)* 5.51 (2.75, 11.05)*	1.35 (0.73, 2.49) 2.39 (1.14, 4.99)*
**B. Age of onset of cannabis use disorder**					
Very early onset (Q1: 12-16y)	131 (1.59)	**3.15 (1.80, 5.49)**	**1.85 (1.00, 3.44)**	**5.51 (2.42, 12.58)**	**2.85 (1.18, 6.89)**
Early onset (Q2:17-18y)	123 (1.79)	1.39 (0.69, 2.81)	1.03 (0.49, 2.15)	**3.08 (1.22, 7.77)**	2.17 (0.84, 5.59)
Middle onset (Q3: 19-20y)	89 (1.26)	1.77 (0.64, 4.95)	1.08 (0.41, 2.84)	1.24 (0.20, 7.66)	0.70 (0.11, 4.46)
Late onset (Q4: 21-50y)	101 (1.09)	**3.38 (1.65, 6.94)**	1.93 (0.92, 4.05)	**3.47 (1.46, 8.24)**	1.73 (0.62, 4.82)
No onset of use disorder	8329 (94.28)	***Reference***	***Reference***	***Reference***	***Reference***
**C. Age of onset of cannabis dependence**					
Very early onset (Q1: 12-16y)	32 (0.38)	**5.83 (2.09, 16.19)**	**4.38 (1.39, 13.79)**	**12.73 (3.73, 43.66)**	**9.41 (2.42, 36.49)**
Early onset (Q2:17-19y)	37 (0.65)	1.18 (0.27, 5.09)	0.76 (0.16, 3.52)	1.86 (0.49, 7.00)	1.11 (0.28, 4.44)
Middle onset (Q3: 20-22y)	28 (0.28)	1.43 (0.37, 5.52)	0.67 (0.16, 2.81)	**-**	-
Late onset (Q4: 23-50y)	31 (0.41)	**10.39 (354, 30.56)**	**4.97 (1.58, 15.64)**	**6.34 (2.28, 17.63)**	2.41 (0.76, 7.63)
No onset	8645 (98.27)	***Reference***	***Reference***	***Reference***	***Reference***

^1^Model 1 = Adjusted for age, sex, ^2^Model 2 = Adjusted for age, sex, marital status, migrant status, employment status, educational status, any anxiety disorders, any depressive disorders, physical disorders and 'other' substance harmful use/dependence ('other' substance harmful use/dependence denotes all other drug or alcohol use/dependence other than cannabis) ^3^OR = Odds Ratio, ^4^CI = 95% Confidence Interval; *significance: *p *< 0.01 (shown in bold) - No estimates because of low sample size

### Association between alcohol use disorders and DLE

Table 5 shows the association between alcohol use disorders and DLE. While 18% of the adult population had lifetime alcohol use disorder, the pattern of association between this exposure and DLE was less consistent across the adjusted models and the exposure categories. Concerning age of onset of these two diagnoses versus DLE, those with early onset (17 years or less) were significantly more likely to endorse DLE probe items, however inspection of the data did not suggest a consistent dose-response across the age of onset categories.

**Table 5:** Relationship between lifetime alcohol use disorder and delusional-like experiences (n = 8,773)

		Any Delusional-like experiences			
		Screen items		Probe items	
A. Lifetime alcohol use/dependence disorder					
	Count (%)	Model 1^1^ OR^4 ^(95% CI^5^)	Model 2^2^ OR^4 ^(95% CI^5^)	Model 1^1^ OR^4 ^(95% CI^5^)	Model 2^2^ OR^4 ^(95% CI^5^)
**Alcohol use disorder, lifetime** **Alcohol dependence disorder, lifetime**	1490 (18.31) 329 (3.71, 0.28)	1.18 (0.89, 1.56) 3.50 (2.33, 5.28)*	1.01 (0.76, 1.33) 1.85 (1.15, 2.97)*	1.41 (0.97, 2.03) 4.48 (2.39, 8.40)*	1.09 (0.72, 1.63) 1.93 (0.91, 4.07)
**B. Age of onset of alcohol use disorder**					
Very early onset (Q1: 9-17y)	361 (4.49)	**2.26 (1.48, 3.46)**	1.54 (0.99, 2.39)	**3.50 (1.79, 6.84)**	**2.06 (1.02, 4.18)**
Early onset (Q2:18-19y)	487 (6.34)	1.03 (0.70, 1.50)	0.87 (0.59, 1.29)	1.40 (0.75, 2.61)	1.14 (0.59, 2.16)
Middle onset (Q3: 20-25y)	509 (6.34)	**1.55 (1.09, 2.19)**	1.19 (0.80, 1.78)	**2.17 (1.35, 3.51)**	1.53 (0.87, 2.67)
Late onset (Q4: 26-79y)	441 (4.58)	**2.25 (1.45, 3.49)**	1.53 (0.96, 2.42)	**2.08 (1.01, 4.29)**	1.26 (0.54, 2.95)
No onset of use disorder	6975 (78.26)	***Reference***	***Reference***	***Reference***	***Reference***
**C. Age of onset of alcohol dependence disorder**					
Very early (Q1: 12-17 y)	66 (0.86)	**3.63 (1.84, 7.17)**	1.69 (0.82, 3.49)	**8.73 (3.33, 22.91)**	**3.51 (1.29, 9.52)**
Early (Q2:18-20y)	100 (1.07)	1.19 (0.66, 2.15)	0.71 (0.39, 1.29)	2.18 (0.84, 5.65)	1.14 (0.45, 2.82)
Middle (Q3: 21-29y)	75 (0.85)	**4.45 (1.79, 11.05)**	1.99 (0.79, 5.02)	**3.23 (1.16, 8.99)**	1.18 (0.36, 3.88)
Late (Q4: 30-79y)	83 (0.84)	**7.12 (2.97, 17.09)**	**4.29 (1.24, 14.89)**	**5.51 (131, 23.27)**	2.84 (0.46, 17.66)
No onset	8449 (96.39)	***Reference***	***Reference***	***Reference***	***Reference***

^1^Model 1 = Adjusted for age, sex, ^2^Model 2 = Adjusted for age, sex, marital status, migrant status, employment status, educational status, any anxiety disorders, any depressive disorders, physical disorders and 'other' substance harmful use/dependence ('other' substance use/dependence disorders denotes all other drug use/dependence other than alcohol) ^3^OR = Odds Ratio, ^4^CI = 95% Confidence Interval; *significance: *p *< 0.01 (shown in bold)

Finally, we undertook several post-hoc analyses in order to explore the robustness of the findings. We examined the relationship between the variables of interest when the harmful use and dependence disorders for both cannabis and alcohol were restricted to past year diagnoses (instead of lifetime ever). The pattern of findings remained unchanged (data not shown). We also examined the association between alcohol use disorders versus DLE when demographic variables such as migrant status, employment status and educational status were removed from Model 2. Again, the pattern of findings remained essentially unchanged (data not shown).

## Discussion

Using a large nationally representative sample, we investigated the association between DLE versus tobacco use, and cannabis and alcohol use disorders. We found that DLE were more likely to be endorsed by those with (a) everyday tobacco use, or (b) smoking onset aged 15 years or younger. In keeping with studies exploring the links between cannabis use and psychotic disorders, we found that DLE were significantly more common in those with (a) cannabis dependence, or (b) onset of cannabis use 16 years or younger. The general pattern of association persisted when adjusted for a wide range of potential confounding factors, including comorbid polysubstance use. These main findings are congruent with previous study results of a similar population sample in Australia [15]. In addition, the current study found that earlier age at onset of tobacco use, and cannabis use disorders were significantly associated with DLE. In contrast, the pattern of association between alcohol use/dependence disorder and DLE was less consistent when adjusted for the presence of other substance use disorders.

### The association between DLE and tobacco use

We found several significant associations between tobacco-related variables and DLE. For example, compared to non-smokers, DLE endorsement was more common in those (a) those who use tobacco every day (b) those who start smoking earlier before the age of 15 years those who used either 16-22 or 23 or more cigarettes per day. These associations remained significant after controlling for a set of potential confounding factors related to DLE and smoking. The results are consistent with several prospective studies in which tobacco smoking was found to be associated with later psychotic symptoms [18-20]. The mechanisms underlying this association remains unclear. Mindful that observational studies are vulnerable to residual confounding, the findings raise several research questions suitable for future study. It is possible that the association between tobaccos use and DLE may reflect an increased propensity of young people with psychotic experiences to commence tobacco use. This may be influenced by intrinsic factors (eg., shared genes) that predispose people with psychotic symptoms to initiate and maintenance of smoking behaviours [34]. It is also feasible that nicotine in cigarettes could modulate mesolimbic dopamine neurotransmitter systems [35,36], which may increase the vulnerability to DLE in susceptible individuals. Animal studies also show that nicotine promotes the release of other neurotransmitters including acetylecholine, endogeneous opioid peptides, GABA, norepinephrine and serotonin [37] which may influence the pathogenesis of psychotic symptoms.

### The association between DLE and cannabis use disorders

In addition to the significant association between DLE and cannabis use disorders, individuals with onset of use disorders aged 16 years or younger had increased odds of endorsing DLEs. While numerous prospective studies have found an independent effect of cannabis use on the development of psychotic disorders [3,4,6,9], our findings are congruent with recent evidence showing that cannabis use at a younger age was associated with psychotic experiences [17]. One possible explanation is that early exposure to THC (an active ingredients of cannabis) during critical periods of brain maturation has an impact on the development of several neurotransmitter systems which may interfere crucial process of brain development [38]. Recent evidence suggests that Delta 9-THC modulates mediotemporal and ventrostriatal function that may impair verbal learning and provoke acute psychosis [39,40]. While it is believed that Delta 9-THC may produce permanent changes in the central nervous system [41], recent studies found a clear improvement in outcome in those who stopped use cannabis after the first psychotic episode [42].

### The association between DLE and alcohol use and use disorders

In contrast to association between tobacco smoking or cannabis use and DLE, the pattern of association between alcohol use disorders and DLE was less consistent when adjusted for the presence of other substance use disorders. For example, alcohol dependence was significantly associated with endorsement of screen items, but not probe items. The lack of association with the probe items may reflect lack of power, as these items were less frequently endorsed. There was some evidence that early onset of alcohol use and dependence disorders were associated with DLE endorsement (most noticeably for probe items). While those with comorbid schizophrenia and alcohol use disorders have worse clinical outcomes [43], alcohol use has not been identified as a risk factor for psychosis. It is feasible that alcohol use may contribute to an abnormal persistence of DLE (i.e. while DLE endorsement usually decreases over age, those who use alcohol may have persistent DLE). This research question may be suitable for further scrutiny within longitudinal prospective studies.

### Limitations

The study had several limitations. Importantly, the study was cross-sectional and it was not possible to establish the direction of causality between DLEs and the measures of substance use (we do not have information on the age-of-onset of the DLE). Using a prospective birth cohort and a nested sibling-pair study, we have previously reported an association between early cannabis use and later DLE and hallucinations [4]. We hope to explore the association between early alcohol and tobacco use and psychosis-related outcomes in this prospective cohort in future studies. With respect to the outcome measure, there were no items for hallucinations. However, previous studies have shown a strong association between DLE and hallucinations in general population samples [44-46]. Finally, because the data were obtained from a household survey some population groups such as homeless, people living in nursing home, hostels etc were not surveyed. The study results may not also be generalised in other countries because of potential differences in cultures and socio-economic structures.

## Conclusion

Our study provides evidence that tobacco, alcohol and cannabis use disorders are associated with an increased risk of endorsing DLEs. The findings confirm previous findings, and contribute to the emerging literature indicating shared risk factors between DLE and psychotic disorders [47,48], and suggest the need for continued research on the potentially harmful effects of the substance use disorders on psychosis-related outcomes.

## Conflicts of interests

The authors declare that they have no competing interests.

## Authors' contributions

JM and SS conceptualized the study, SS supervised the data analyses, All authors participated in the interpretation of the data and the writing of the manuscript. All authors read and approved the final manuscript.

## Pre-publication history

The pre-publication history for this paper can be accessed here:

http://www.biomedcentral.com/1471-244X/11/202/prepub
